# Compensation for intracellular environment in expression levels of mammalian circadian clock genes

**DOI:** 10.1038/srep04032

**Published:** 2014-02-07

**Authors:** Ritsuko Matsumura, Akihiko Okamoto, Koichi Node, Makoto Akashi

**Affiliations:** 1 The Research Institute for Time Studies, Yamaguchi University, 1677-1 Yoshida, Yamaguchi 753-8511, Japan; 2 Department of Cardiovascular Medicine, Saga University, 5-1-1 Nabeshima, Saga 849-8501, Japan

## Abstract

The circadian clock is driven by transcriptional oscillation of clock genes in almost all body cells. To investigate the effect of cell type-specific intracellular environment on the circadian machinery, we examined gene expression profiles in five peripheral tissues. As expected, the phase relationship between expression rhythms of nine clock genes was similar in all tissues examined. We also compared relative expression levels of clock genes among tissues, and unexpectedly found that quantitative variation remained within an approximately three-fold range, which was substantially smaller than that of metabolic housekeeping genes. Interestingly, circadian gene expression was little affected even when fibroblasts were cultured with different concentrations of serum. Together, these findings support a hypothesis that expression levels of clock genes are quantitatively compensated for the intracellular environment, such as redox potential and metabolite composition. However, more comprehensive studies are required to reach definitive conclusions.

Almost all living organisms exhibit circadian rhythms in physiology and behavior^1^,^2^. These rhythms are driven by the internal clock, which enables adaptational synchrony with the earth's rotation period. The central circadian clockwork consists of a negative feedback loop of transcription. Under this, expression of the *Period* (*Per*) genes is driven by the CLOCK (NPAS2)/BMAL1 transcription complex. Subsequently, the PER proteins, together with Cryptochrome (CRY), serve to negatively regulate the CLOCK (NPAS2)/BMAL1 transcription complex^3^,^4^. DBP functions as a stabilizer of the machinery^5^. In addition to this core machinery, ROR and REV-ERB proteins generate *Bmal1* expression rhythms which enhance the robustness of the core feedback system^6^,^7^. As a representative example of circadian robustness, it is well known that circadian gene expression is compensated for temperature: even when cells are cultured at different temperatures, only minor effects on circadian expression pattern are observed^8^.

Cell-autonomous circadian oscillation of clock gene expression is observed in almost all tissues throughout the body^9^. However, while gene expression profiles, metabolite composition, and redox potential vary depending on cell type, and the intracellular environment accordingly shows wide variation in a cell type-specific manner, it remains unclear how the core clock machinery is maintained against the variable intracellular environment.

Here, to better understand the maintenance and control of the core clock machinery across different intracellular environments, we compared the temporal expression pattern of clock genes among a range of peripheral tissues. We also examined the change in circadian gene expression in response to different nutritional conditions.

## Results

**Figure 1:**
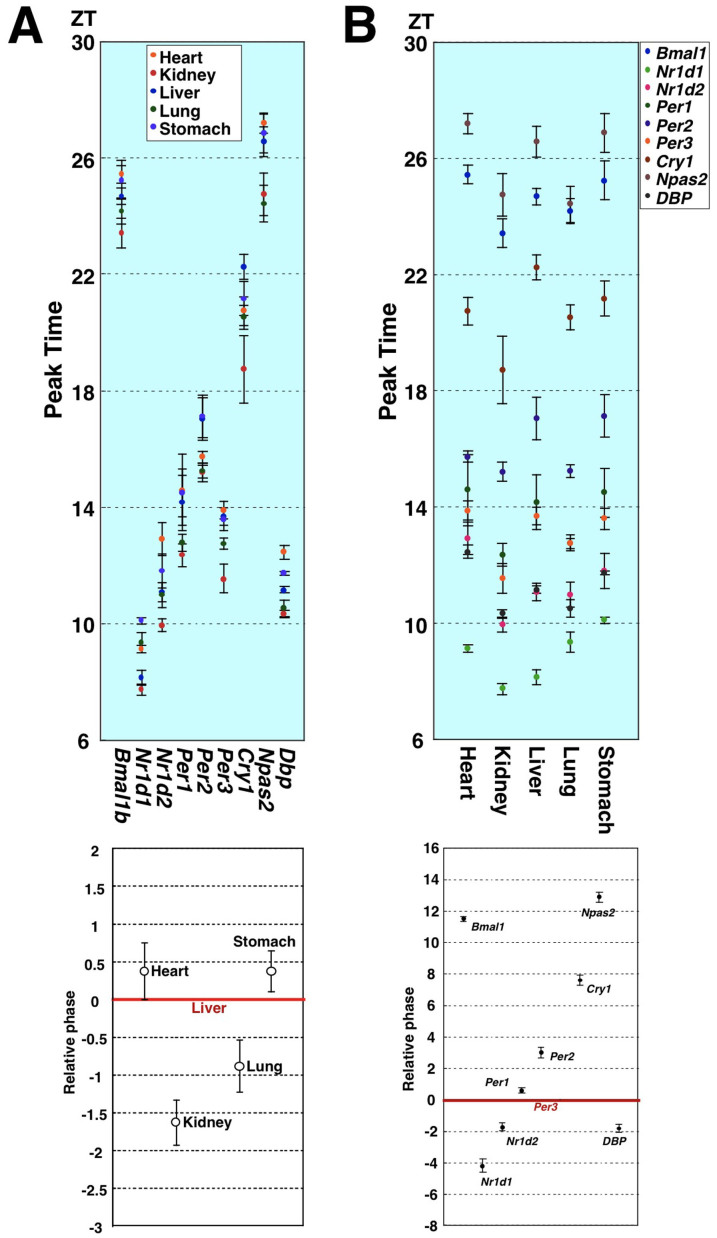
Difference and similarity in circadian phase of clock gene expression between five mouse peripheral tissues. Circadian expression of nine clock genes in five mouse peripheral tissues was measured by real-time PCR. Relative levels of mRNA were normalized to the corresponding *18S-rRNA* levels. The peak times calculated by cosine curve-fitting were compared. Each value represents the average of three independent RT-PCR experiments. (A) Upper panel: Phase comparison among the five mouse peripheral tissues for the nine clock genes. Lower panel: To determine the relative phase of these peripheral clocks, circadian phase relative to the liver was calculated for each clock gene, and these phase intervals were then averaged. (B) Upper panel: Phase relations among the nine clock genes in the five mouse peripheral tissues. Lower panel: The circadian phases of the nine clock genes relative to that of *Per3* were calculated for each peripheral tissue, and these phase differences were then averaged.

To investigate the presence of cell type-dependent differences in clock machinery, we compared clock gene expression properties between peripheral tissues. Hence, we examined the circadian expression of nine clock genes in five mouse peripheral tissues, and compared their circadian peak times as deduced by cosine curve-fitting (Fig. 1A, upper panel). Our findings showed that the acrophases were similar in all tissues examined, regardless of the type of clock gene examined. Next, to determine the relative phases of these peripheral clocks, we calculated the circadian phase relative to the liver for each clock gene, and then averaged these phase intervals (Fig. 1A, lower panel). We found that the phase differences among these peripheral clocks were within approximately 2 hours, indicating that the phase of peripheral circadian clocks are somewhat tissue-specific but synchronized within a similar range *in vivo*. As another feature, phase intervals among these nine clock genes appeared to be strongly fixed regardless of the type of peripheral tissue (Fig. 1B, upper panel). This finding makes sense, if it is correct that every peripheral clock is driven by exactly the same components and mechanism. We then calculated the acrophase of the nine clock genes relative to that of *Per3* for each peripheral tissue (the *Per3* phase was set to 0), and averaged these phase differences (Fig. 1B, lower panel). The phase relationship was completely conserved in every one of these five peripheral clocks.

**Figure 2:**
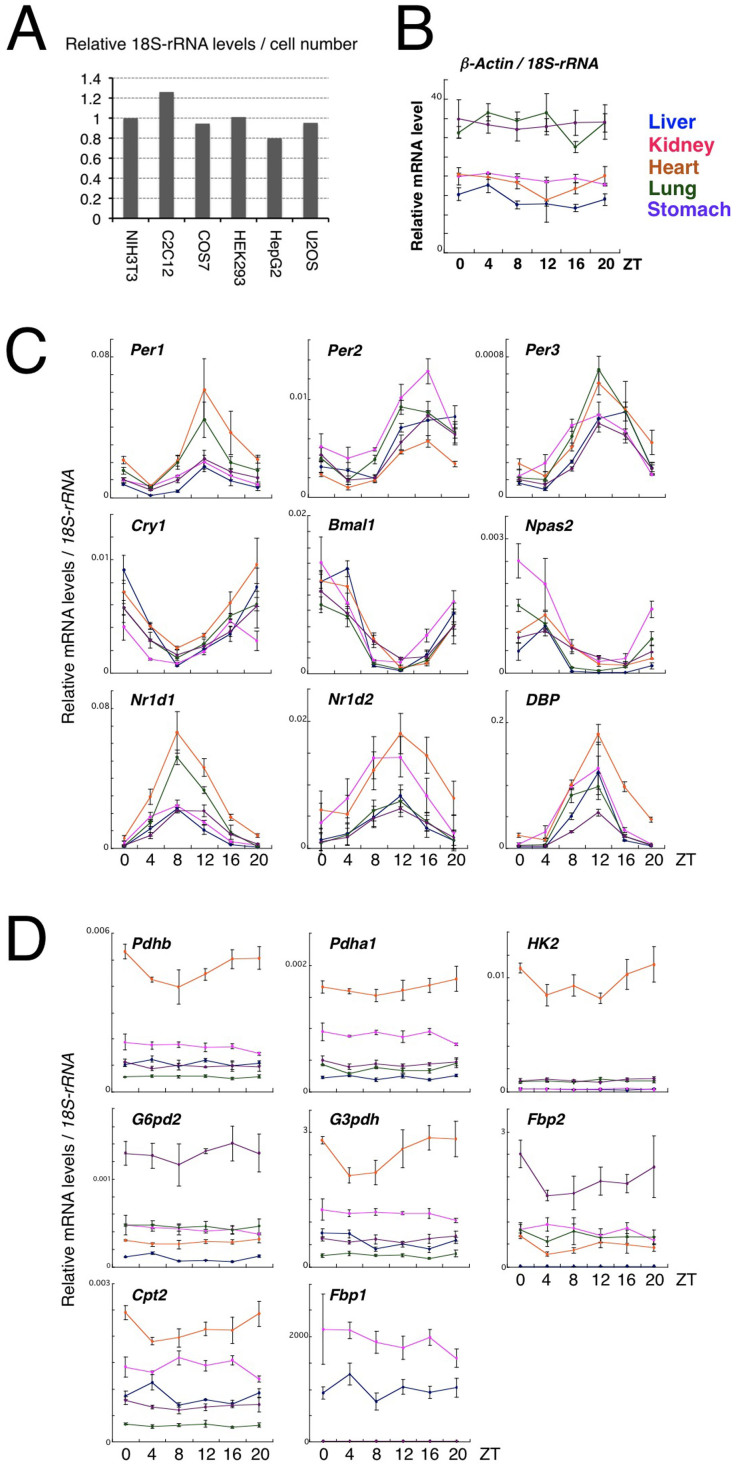
Cell type-independent expression intensity of clock genes. (A) *18S-rRNA* expression levels per cell number in NIH3T3 (mouse fibroblasts), C2C12 (mouse skeletal myocytes), COS7 (SV40-transformed monkey kidney), HEK293 (human embryonic kidney cells), HepG2 (human hepatoma cells) and U2OS (human osteosarcoma cells). The value in NIH3T3 was set to 1. The data are the average of two independent experiments. (B, C and D) The *Zeitgeber* times (ZT) at which three animals were sacrificed are given on the abscissas of the top diagram. Diurnal variation in expression of *beta-Actin* (B), clock genes (C), and metabolism-related housekeeping genes (D) in the five peripheral tissues are shown. Expressions relative to *18S-rRNA* are shown. Gene abbreviations: *Pdhb*, pyruvate dehydrogenase beta; *Pdha1*, pyruvate dehydrogenase alpha 1; *HK2*, hexokinase 2; *G6pd2*, glucose-6-phosphate dehydrogenase 2; *G3pdh*, glycerol-3-phosphate dehydrogenase; *Fbp2*, fructose-1,6-bisphosphatase 2; *Cpt2*, carnitine palmitoyl transferase 2; and *Fbp1*, fructose-1,6-bisphosphatase 1.

Next, we tried to compare clock gene expression levels per cell number between different tissues. However it is almost impossible to completely disperse tissues and count the cell number unlike in the case of cultured cells. We therefore decided to use expression levels of ribosomal RNA as a normalizing control for cell number, which are very high and stable compared with the other genes. Indeed, *18S-rRNA* levels per cell number varied within a small range (approx. 1.5-fold) when compared among NIH3T3 (mouse fibroblasts), C2C12 (mouse skeletal myocytes), COS7 (SV40-transformed monkey kidney), HEK293 (human embryonic kidney cells), HepG2 (human hepatoma cells) and U2OS (human osteosarcoma cells), supporting the hypothesis that the gene is expressed at a similar level independently of cell types (Fig. 2A). We confirmed that *18S-rRNA*-normalized expression levels of *beta-Actin*, a housekeeping gene commonly used as an internal control, varied less than three-fold between tissues (Fig. 2B). Interestingly, when we compared 18S-rRNA-normalized levels of expression of the clock genes among the five peripheral tissues (Fig. 2C), we found that the differences in expression levels varied up to approximately three-fold. Particularly in the case of core clock components such as *Per2*, *Bmal1* and *Cry1*, the fold variation was only up to about two-fold. This extent of variation was similar to that of *beta-Actin*, a general housekeeping gene (Fig. 2B). In the case of 18S-rRNA-normalized levels of expression of eight rate-limiting enzymes required for several metabolic processes, in contrast, levels of expression, even of well-known housekeeping genes such as *G3pdh* and *G6pd2*, exhibited marked variability among the five peripheral tissues (Fig. 2D). These findings strongly suggest that levels of transcription of clock genes are well compensated across a variety of cell types, implying that normal clock function requires quantitative correlation among clock gene products.

**Figure 3:**
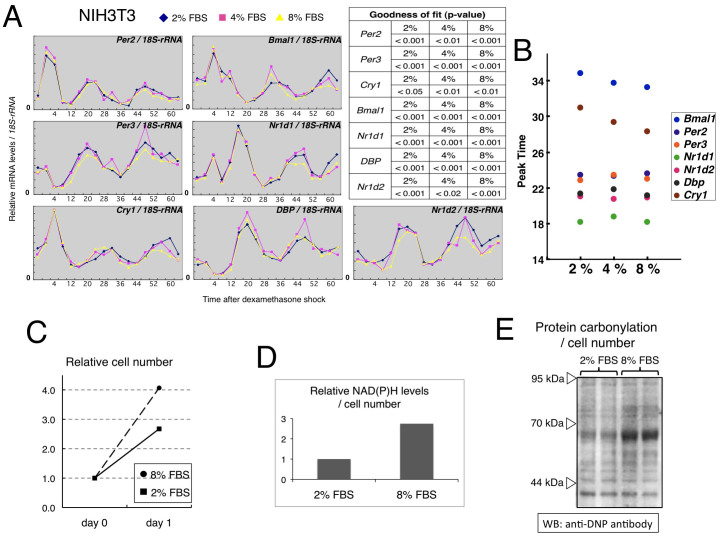
Metabolic activity-independent expression rhythms of clock genes. (A) After synchronization using dexamethasone (DEX, 100 nM for 1 hr), NIH3T3 cells were cultured in the presence of 2%, 4%, or 8% serum, and chronological changes in the expression of the clock genes were compared. Expressions relative to *18S-rRNA* are shown. Goodness of fit values are shown as a table on the upper right (All of them are statistically significant). (B) Comparison of second peak times calculated by cosine curve-fitting. The data show representative results from three experiments. (C, D and E) 24 hrs after exposure of NIH3T3 cells to 2% or 8% FBS-containing medium, cell proliferation, reducing power (NADH and NADPH) and reactive oxygen levels (carbonyl protein) were examined. Raw data of NAD(P)H levels were normalized to cell number. For comparison of protein carbonylation levels, loading amounts of duplicated samples were adjusted to ensure an equal number of cells per lane. The data in (C) and (D) are the average of two independent experiments.

Furthermore, as suggested by tissue-dependent levels of expression of metabolic rate-limiting enzymes, metabolic status differed substantially among these tissues, suggesting that the circadian patterns of clock gene expression are not affected by metabolic conditions. To test this hypothesis further, we cultured dexamethasone-synchronized NIH3T3 cells in the presence of 2%, 4%, or 8% serum and investigated the effects of serum levels on circadian patterns of clock gene expression (Fig. 3A). Generally, serum concentrations affect cellular metabolism and physiological characteristics such as proliferation. We performed a cosinor analysis on the clock gene expression rhythms, and compared the average of the period, phase angle, amplitude and mesor between the 8% and 2% serum conditions. The phase angle (8% − 2%), period (8% − 2%), amplitude (8%/2%) and mesor (8%/2%) were 17° (95% CI: 1.3–33), −2.0 h (95% CI: −3.4–−0.54), 0.90 fold (95% CI: 0.80–1.0) and 0.92 fold (95% CI: 0.87–0.97), respectively; on average, the differences were less than 10%. Moreover, the phase intervals between the clock gene expression rhythms were not largely affected by serum concentration (Fig. 3B). Together, the circadian properties of clock gene expression were stable during exposure to different concentrations of serum (note that the exposure was begun immediately after synchronization of cellular clocks). In addition, the phase intervals of clock genes in NIH3T3 cells were quite similar to those in peripheral tissues, as shown in Fig. 1B (except for *Per2* as noted below), demonstrating that the phase relation among clock genes is maintained in a cell-autonomous fashion. Interestingly, the phase difference between *Per2* and *Per3* was about 3 hours in peripheral tissues, whereas the phases of these two genes were almost identical in NIH3T3 cells. This indicates that the phase of *Per2* expression is regulated not only cell-autonomously but also in a systemic fashion^10^. To confirm the difference of intracellular physiological activities under different concentrations of serum, we examined cell proliferation, reducing power (NADH and NADPH) and reactive oxygen levels (carbonyl protein) 24 hrs after exposure of NIH3T3 cells to 2% or 8% FBS-containing medium (Figs. 3C, 3D and 3E). In all the three experiments, we found distinct differences between two conditions, illustrating that the exposure period was sufficient to affect intracellular physiological activities. However, it would be still possible that some effects of serum concentration on circadian gene expression become obvious after further incubation, but unfortunately it is technically difficult to keep cells alive for more than three days under the experimental condition.

Taken together, our findings suggest that the circadian properties of clock gene expression such as phase relationships and absolute levels of expression are tightly modulated so that they remain independent of cell-type-specific intracellular environment and metabolic state.

## Discussion

The results of this study provide a novel insight into the well-compensated system of the circadian clock. A hallmark characteristic of biological clocks is their ability to minimize the influence of environmental fluctuation in realizing robust autonomous oscillation, which is indispensable for stabilized clockwork. Temperature compensation is a highly impressive and symbolic feature of the circadian clock whose mechanism is still little understood. In addition to temperature, circadian gene expression is surrounded by diverse intracellular environmental factors, which vary widely among different cell types. Indeed, metabolic rate-limiting enzymes show tissue-dependent levels of expression as demonstrated in Fig. 2. Interestingly, in contrast, our present findings suggest that circadian gene expression profiles such as phase, amplitude and expression level are somewhat tissue-specific but not largely affected by cell type-specific intracellular environment. Their fluctuation range of expression is smaller than that of the metabolic housekeeping genes examined, indicating that the circadian clock has a specific cell-autonomous compensation system for maintaining constant expression levels. However, further comprehensive studies are required to make definitive conclusions because a limited number of clock and clock-related genes were examined in a limited number of peripheral tissues in the present study.

We are presently unable to explain the mechanism of the quantitative compensation identified here. A study on the single-cell monitoring of *Bmal1* promoter-driven luciferase activity in confluent NIH3T3 fibroblasts that we also used in Fig. 3 reported that luminescence levels were largely affected by exposure to different concentrations of serum^11^, suggesting that quantitative compensation of clock gene transcripts might be the result of posttranscriptional regulation. Indeed, the existence of specific posttranscriptional machinery for quantitative regulation is indicated by the fact that PER2 protein levels show circadian oscillation even when the expression is driven by a constitutive promoter^12^.

The ability to compensate for the intracellular environment might provide valuable information in diagnostic evaluation of the human circadian clock. Among the very few biopsy tissues now available for noninvasive investigation of the human clock are white blood cells, oral mucosa and hair follicle cells, but the compensated circadian gene expression we show here might ensure that any peripheral tissue can be used.

## Methods

### Animals

Mice were housed using a strict 12:12 hour light/dark regimen. During the dark period, mice were sacrificed by cervical dislocation and beheading under dim light. Five peripheral tissues were immediately frozen in liquid nitrogen and stored at −80°C until processed for purification of RNA. After homogenizing frozen tissues (heart, kidney, liver, lung and stomach) in RLT lysis buffer, RNA purification was performed with the RNeasy Mini Kit (Qiagen). All the protocols for animal experiments have been approved in the Animal Research Committee of Yamaguchi University. Animal studies were performed in compliance with the Yamaguchi University Animal Care and Use guidelines.

### mRNA determination

Total RNA was reverse-transcribed using ReverTraAce (Toyobo), and real-time PCR was performed using SYBR Green (ABI) and a 1/20 volume of the reverse transcription product. Data were obtained using PRISM7300 (ABI) and corrected by expression levels of *18S-rRNA*. Primers were selected when there was no unspecific amplification in dissociation curves and when amplification efficiency was relatively favorable. A 24-h period cosine curve was fitted to time-course gene expression data we obtained using a non-linear least-squares method.

### Clock gene expression under different concentrations of serum

NIH3T3 cells were cultured in DMEM supplemented with 10% fetal bovine serum (FBS) and antibiotics (penicillin and streptomycin) at 37°C under 5% CO2. The cells reached confluence 2 days after plating. They were kept for about 24 hrs in medium containing 1% FBS and then subject to a 100 nM dexamethasone shock (t = 0). They were then transferred to medium containing different concentrations of serum (2%, 4% and 8%), immediately frozen in liquid nitrogen at the indicated time points, and stored at −80°C until processed for purification of RNA.

### Statistics

To assess the presence or absence of significant circadian rhythmicity in the NIH3T3 expression data, we performed a curve-fitting procedure and calculated the index of goodness of fit; smaller index values reflect a more fitted curve. To do this, expression data were analyzed with the software Acro, which was provided by Dr. Refinetti. P-values of <0.05 are considered statistically significant.

To evaluate the effect of serum concentration, we performed a cosinor analysis on expression rhythms of the seven clock genes, and compared the period, phase angle, amplitude and mesor between the 8% and 2% serum conditions. The average of the differences and their 95% confidence intervals (CI) were calculated. We used the software Cosinor, which was provided by Dr. Refinetti.

### Detection of NAD(P)H and protein carbonylation

24 hrs after exposure of NIH3T3 cells to 2% or 8% FBS-containing medium, NAD(P)H-Glo Detection System (Promega) was used to detect NADH and NADPH levels. Raw data were normalized to cell number. Protein carbonylation levels were detected by using Protein carbonyls western blot detection kit (Shima laboratories). Loading amounts of samples were adjusted to ensure an equal number of cells per lane.

## Author Contributions

M.A. designed the research. M.A. and R.M. performed the research. A.O. analyzed data. R.M., A.O. and K.N. contributed reagents/materials/analysis tools. M.A. wrote the paper.
